# Lagrangian Fibrations

**DOI:** 10.1007/s00032-022-00349-y

**Published:** 2022-03-19

**Authors:** D. Huybrechts, M. Mauri

**Affiliations:** 1 Mathematisches Institut, Universität Bonn, Endenicher Allee 60, 53115 Bonn, Germany; 2 Max Planck Institute for Mathematics, Vivatsgasse 7, 53111 Bonn, Germany

## Abstract

We review the theory of Lagrangian fibrations of hyperkähler manifolds as initiated by Matsushita. We also discuss more recent work of Shen–Yin and Harder–Li–Shen–Yin. Occasionally, we give alternative arguments and complement the discussion by additional observations.

Assume $f:X\rightarrow B$ is a Lagrangian fibration of a compact hyperkähler manifold *X* of complex dimension 2*n*, and $\pi :{\mathcal {X}} \rightarrow \Delta$ is a type III degeneration of compact hyperkähler manifolds of complex dimension 2*n*. Then the cohomology algebra of ${\mathbb {P}}^n$ appears naturally in (at least) four different guises:

(i) As the cohomology algebra of (0, *p*) resp. (*p*, 0)-forms (both independent of *f*):
  $$\begin{aligned} H^*({\mathbb {P}}^n,{\mathbb {C}})\simeq H^*(X,{{\mathcal {O}}}_X)\quad \text {and}\quad H^*({\mathbb {P}}^n,{\mathbb {C}})\simeq H^0(X,\Omega ^*_X). \end{aligned}$$
(ii) As the cohomology of the base of the fibration:^1^
  $$\begin{aligned} H^*({\mathbb {P}}^n,{\mathbb {C}})\simeq H^*(B,{\mathbb {C}}). \end{aligned}$$
(iii) As the image of the restriction to the generic fiber $X_t$ of *f*:
(iv) As the cohomology of the dual complex ${\mathcal {D}}({\mathcal {X}}_0)$ of the central fiber ${\mathcal {X}}_0$ of $\pi$:
  $$\begin{aligned} H^*({\mathbb {P}}^n,{\mathbb {C}})\simeq H^*({\mathcal {D}}({\mathcal {X}}_0), {\mathbb {C}}). \end{aligned}$$

In this survey we discuss these four situations and explain how they are related. We start by reviewing basic results on Lagrangian fibrations in Sect. 1, discuss the topology of the base and the restriction to the fiber in Sect. 2, and then explain in Sect. 3 how the various occurrences of ${\mathbb {P}}^n$ are related, by sketching the proof of a key identity called P $=$ W.

Throughout, *X* denotes a compact hyperkähler manifold of complex dimension 2*n*. A fibration of *X* is a surjective morphism  with connected fibers onto a normal variety *B* with $0<\dim (B)<2n$. A submanifold $T\subset X$ of dimension *n* is Lagrangian if the restriction $\sigma |_T\in H^0(T,\Omega _T^2)$ of the holomorphic two-form $\sigma \in H^0(X,\Omega _X^2)$ is zero.

## Basics on Lagrangian Fibrations

We first discuss Lagrangian submanifolds and in particular Lagrangian tori. Then we study the cohomology and the singularities of the base *B*. Next we show that the fibers, smooth ones as well as singular ones, of any fibration are Lagrangian and conclude that fibrations of hyperkähler manifolds over a smooth base are flat.

At the end, we mention further results and directions without proof: Matshushita’s description of the higher direct image sheaves $R^if_*{{\mathcal {O}}}_X$, Beauville’s question whether Lagrangian tori are always Lagrangian fibers, smoothness of the base, etc.

### Lagrangian Tori

We start with some general comments on Lagrangian manifolds and more specifically on Lagrangian tori.

#### Proposition 1.1

(Voisin) Any Lagrangian submanifold $T\subset X$ of a hyperkähler manifold *X* is projective. In particular,  any Lagrangian torus is an abelian variety.

#### Proof

We follow the proof as presented in [11]. Since the restriction of any Kähler class on *X* to *T* is non-trivial, the restriction  is a non-trivial morphism of Hodge structures. On the other hand, as *T* is Lagrangian, all classes in $H^{2,0}(X)\oplus H^{0,2}(X)$ have trivial restrictions. Hence, the image of  is contained in $H^{1,1}(T,{\mathbb {R}})$. More precisely, the images of  and of  coincide. Therefore, for any Kähler class $\omega \in H^{1,1}(X,{\mathbb {R}})$ there exists a rational class $\alpha \in H^2(X,{\mathbb {Q}})$ such that the (1, 1)-class $\alpha |_T$ comes arbitrarily close to the Kähler class $\omega |_T$. Thus, $\alpha |_T$ is a rational Kähler class and, hence, *T* is projective.$\square$

#### Remark 1.2

The normal bundle of a Lagrangian submanifold $T\subset X$ is isomorphic to the cotangent bundle of *T*, so ${\mathcal {N}}_{T/X}\simeq \Omega _T$. Hence, the (1, 1)-part of the restriction map  can be identified with the natural map  that sends a first order deformation of *X* to the obstruction to deform *T* sideways with it, see [69]:Clearly, as *T* is Lagrangian, the map  is trivial, see the proof above. Since the restriction of a Kähler class is again Kähler,  is certainly not trivial. Thus, $T\subset X$ deforms with *X* along a subset of codimension at least one. For smooth fibers of a Lagrangian fibration, so eventually Sect. 1.5.2 for all Lagrangian tori, the rank of the restriction map and hence the codimension of the image  is exactly one.^2^

#### Proposition 1.3

Assume $T\subset X$ is a Lagrangian torus. Then the restrictions $\mathrm{c}_i(X)|_T\in H^{2i}(T,{\mathbb {R}})$ of the Chern classes $\mathrm{c}_i(X)\in H^{2i}(X,{\mathbb {R}})$ are trivial.

#### Proof

The normal bundle sequence allows one to compute the restriction of the total Chern class of *X* to *T*. More precisely, $\mathrm{c}({\mathcal {T}}_X)|_T=\mathrm{c}({\mathcal {T}}_T)\cdot \mathrm{c}({{\mathcal {N}}}_{T/X})$. To conclude, use ${{\mathcal {N}}}_{T/X}\simeq \Omega _T$ and the fact that the tangent bundle of a torus is trivial. $\square$

#### Remark 1.4

(i) In the case when $T\subset X$ is the fiber of a Lagrangian fibration , as it always is, see Sect. 1.5.2, the restriction of the Beauville–Bogomolov–Fujiki form, thought of as a class ${\tilde{q}}\in H^4(X,{\mathbb {Q}})$, is also trivial:

$$\begin{aligned} {\tilde{q}}|_T=0. \end{aligned}$$

There does not seem to be a direct proof of this fact. However, using that the rank of the restriction map  is one, see Theorem 2.1, it can be shown as follows. The classes ${\tilde{q}}$ and $\mathrm{c}_2$ in $H^4(X,{\mathbb {Q}})$ both have the distinguished property that the homogenous forms $\int _X{\tilde{q}}\cdot \beta ^{2n-2}$ and $\int \mathrm{c}_2(X)\cdot \beta ^{2n-2}$ defined on $H^2(X,{{\mathbb {Z}}})$ are non-trivial scalar multiples of $q(\beta )^{n-1}$ and, therefore, of each other.^3^ If $[T]\in H^{2n}(X,{{\mathbb {Z}}})$ is the class of a fiber $f^{-1}(t)$, then up to scaling $[T]=f^*\alpha ^n$ for some $\alpha \in H^2(B,{\mathbb {Q}})$. Hence, for a Kähler class $\omega$ on *X* we find (up to a non-trivial scalar factor)

$$\begin{aligned}&\int _T{\tilde{q}}|_T\cdot \omega |_T^{n-2}=\int _X{\tilde{q}}\cdot f^*\alpha ^n\cdot \omega ^{n-2}=\int _X\mathrm{c}_2(X) \cdot f^*\alpha ^n\cdot \omega ^{n-2}\\&\quad =\int _T\mathrm{c}_2(X)|_T\cdot \omega |_T^{n-2}=0. \end{aligned}$$

Since $\omega |_T\ne 0$ and  is generated by $\omega |_T$, this proves the claim.

(ii) For other types of Lagrangian submanifolds, the restrictions of the Chern classes of *X* are not trivial. For example, for a Lagrangian plane ${\mathbb {P}}^2\subset X$ one easily computes $\int _{{\mathbb {P}}^2}\mathrm{c}_2(X)|_{{\mathbb {P}}^2}=15$.

As remarked before, the normal bundle of a Lagrangian torus is trivial. The next observation can be seen as a converse, it applies in particular to the smooth fibers of any fibration .

#### Lemma 1.5

Assume $T\subset X$ is Lagrangian submanifold with trivial normal bundle. Then *T* is a complex torus and,  therefore,  an abelian variety.

#### Proof

Since *T* is Lagrangian, the tangent bundle ${\mathcal {T}}_T\simeq {{\mathcal {N}}}^*_{T/X}$ is trivial. Using the Albanese morphism, one easily proves that any compact Kähler manifold with trivial tangent bundle is a complex torus. $\square$

### The Base of a Fibration

We pass on to (Lagrangian) fibrations.

#### Proposition 1.6

(Matsushita) Assume  is a fibration with *B* smooth. Then *B* is a simply connected,  smooth projective variety of dimension *n* satisfying $H^{p,0}(B)=H^{0,p}(B)=0$ for all $p>0$ and $H^2(B,{\mathbb {Q}})\simeq {\mathbb {Q}}$. In particular, 

$$\begin{aligned} \mathrm{Pic}(B)\simeq H^2(B,{{\mathbb {Z}}})\simeq {{\mathbb {Z}}}. \end{aligned}$$

#### Proof

The smoothness of *B* implies that the pull-back  is injective; see Remark 1.14. Next, as $\alpha ^{2n}=0$ for any class $\alpha \in H^2(B,{\mathbb {R}})$, we have $(f^*\alpha )^{2n}=0$ and, therefore, $q(f^*\alpha )=0$. By [9, 68], this implies $(f^*\alpha )^{n+1}=0$ and hence $\alpha ^{n+1}=0$, which implies $\dim (B)\le n$. On the other hand, again by [9, 68], $(f^*\alpha )^n\ne 0$ for every nonzero class $\alpha \in H^2(B,{\mathbb {R}})$ from which we deduce $n\le \dim (B)$.

If $\alpha \in H^{p,0}(B)$, then $f^*\alpha$ is a non-trivial multiple of some power of $\sigma$. Hence, $\alpha =0$ if *p* is odd. If $p=2$, then $f^*\alpha =\lambda \cdot \sigma$ and, hence, $f^*\alpha ^n=\lambda ^n\cdot \sigma ^n$. Since $\sigma ^n\ne 0$ and $H^{2n,0}(B)=0$, one finds $\lambda =0$. A similar argument can be made to work for all even *p* and an alternative argument is provided by Theorem 2.1.

Next we show $H^2(B,{\mathbb {Q}})\simeq {\mathbb {Q}}$. Using [9, 68], we have

$$\begin{aligned} S^n f^*H^2(B,{\mathbb {Q}})\subset S^nH^2(X,{\mathbb {Q}})\subset H^{2n}(X,{\mathbb {Q}}). \end{aligned}$$

On the other hand, the image of $S^nf^*H^2(B,{\mathbb {Q}})$ is contained in $f^*H^{2n}(B,{\mathbb {Q}})$ which is just one-dimensional.^4^

Since *X* is Kähler, so is *B*, see [67]. Using $H^{2,0}(B)=H^{0,2}(B)=0$, we can conclude that there exists a rational Kähler class on *B*. Hence, *B* is projective. According to [42, Prop. 2.10.2], the natural map  is surjective and, therefore, *B* is simply connected, as *X* is.^5^ Then, by the universal coefficient theorem, $H^2(B,{{\mathbb {Z}}})$ is torsion-free, i.e. $H^2(B,{{\mathbb {Z}}})\simeq {{\mathbb {Z}}}$. Since $H^{1,0}(B)=H^{2,0}(B)=0$, the exponential sequence gives . $\square$

#### Remark 1.7

In fact, as we shall see, $H^{p,q}(B)=0$ for all $p\ne q$ and $H^{p,p}(B)\simeq H^{p,p}({\mathbb {P}}^n)$, i.e. there is an isomorphism of rational Hodge structures

$$\begin{aligned} H^*(B,{\mathbb {Q}})\simeq H^*({\mathbb {P}}^n,{\mathbb {Q}}). \end{aligned}$$

There are two proofs of this fact, both eventually relying on the isomorphism $H^*(X,{{\mathcal {O}}}_X)\simeq H^*({\mathbb {P}}^n,{\mathbb {C}})$. It seems that unlike $H^2(B,{\mathbb {Q}})\simeq {\mathbb {Q}}$, which was proved above by exploiting the structure of the subring of $SH^2(X,{\mathbb {Q}})\subset H^*(X,{\mathbb {Q}})$, the proof of the identities $H^{k}(B, {\mathbb {Q}})\simeq H^{k}({\mathbb {P}}^n, {\mathbb {Q}})$ for $k>2$ uses deeper information about the hyperkähler structure.

(i) The first proof for *B* smooth and *X* projective was given by Matsushita [51], as a consequence of the isomorphisms $R^if_*{{\mathcal {O}}}_X\simeq \Omega _B^i$, see Sect. 1.5.1. Combining this isomorphism with the splitting $Rf_*{{\mathcal {O}}}_X\simeq \bigoplus R^if_*{{\mathcal {O}}}_X[-i]$, see [41], one finds  which proves the claim.^6^
(ii) Another one, which also works for singular *B* and non-projective *X*, was given in [66] and roughly relies on the fact that $H^*(B,{\mathbb {C}})$ can be deformed into $H^*(X,{{\mathcal {O}}}_X)$, see Sect. 2.2.

#### Lemma 1.8

(Markushevich, Matsushita) Under the above assumptions,  *B* is a Fano variety,  i.e. $\omega _B^*$ is ample.

#### Proof

Since *B* is dominated by *X*, we have $\mathrm{kod}(B)\le 0$ by the known case of the Iitaka conjecture; see [36, Cor. 1.2]. Hence, $\omega _B\simeq {{\mathcal {O}}}_B$ or $\omega _B^*$ is ample. However, the first case is excluded by $H^{n,0}(B)=0$.

In [32, Prop. 24.8] the assertion is deduced from the fact that *X* admits a Kähler–Einstein metric. The case $\omega _B\simeq {{\mathcal {O}}}_B$ is excluded, because it would imply $H^{n,0}(B)\ne 0$, which was excluded above. $\square$

#### Remark 1.9

It turns out that as soon as the base *B* is smooth, then $B\simeq {\mathbb {P}}^n$. This result is due to Hwang [34] and its proof relies on the theory of minimal rational tangents. The results by Matsushita and more recently by Shen and Yin, see Remark 1.7 and Sect. 2, can be seen as strong evidence for the result. In dimension two, the result is immediate: Any smooth projective surface *B* with $\omega _B^*$ ample and $H^2(B,{\mathbb {Q}})\simeq {\mathbb {Q}}$ is isomorphic to ${\mathbb {P}}^2$.

It is tempting to try to find a more direct argument in higher dimension, but all attempts have failed so far. For example, according to Hirzebruch–Kodaira [29] it suffices to show that $H^*(B,{{\mathbb {Z}}})\simeq H^*({\mathbb {P}}^n,{{\mathbb {Z}}})$ such that the first Chern class of a line bundle *L* corresponding to a generator of $H^2(B,{{\mathbb {Z}}})$ satisfies $h^0(B,L^k)=h^0({\mathbb {P}}^n,{{\mathcal {O}}}(k))$, see [47] for a survey of further results in this direction.

Alternatively, by Kobayashi–Ochai [39], it is enough to show that $\omega _B$ is divisible by $n+1$, i.e. the Fano manifold *B* has index $n+1$. As a first step, one could try to show that $f^*\omega _B$ is divisible by $n+1$.

### Singularities of the Base

It is generally expected that the base manifold *B* is smooth, but at the moment this is only known for $n\le 2$, see [7, 35, 61]. The expectation is corroborated by the following computations of invariants of the singularities of *B*.

Denote by $I\!H^*(B, {\mathbb {Q}})$ the intersection cohomology of the complex variety *B* with middle perversity and rational coefficients. It is the hypercohomology of the intersection cohomology complex ${\mathcal {IC}}_B$, i.e. $I\!H^*(B, {\mathbb {Q}}) = {\mathbb {H}}^*(B, {\mathcal {IC}}_B)$. In particular, if *B* is smooth or has quotient singularities, see [24, Prop. 3], then $I\!H^*(B, {\mathbb {Q}}) = H^*(B, {\mathbb {Q}})$.

#### Proposition 1.10

Assume  is a fibration over the complex variety *B*.

(i) *B* is ${\mathbb {Q}}$-factorial, 7 both in the Zariski and in the analytic topology.
(ii) The intersection cohomology complex ${\mathcal {IC}}_B$ of *B* is quasi-isomorphic to the constant sheaf ${\mathbb {Q}}_B$. In particular,  $I\!H^*(B, {\mathbb {Q}})=H^*(B, {\mathbb {Q}})$.
(iii) *(Matsushita)* *B* has log terminal singularities.

#### Proof

For (i) and (ii) one only needs that  is a connected and equidimensional morphism from a smooth variety *X*, while in the proof of (iii) one also needs $\omega _X$ trivial.

For any $t \in B$, choose a chart , centered at *x*, and the analytic subset $S {:}{=}\varphi ^{-1}(\Lambda )$, where $\Lambda \subseteq {\mathbb {C}}^{2n}$ is an *n*-dimensional affine subspace intersecting the fiber $\varphi (f^{-1}(t))$ transversely. Since *f* is equidimensional, the restriction  is finite over an analytic neighbourhood *U* of *t*. Therefore, *U* is ${\mathbb {Q}}$-factorial by [38, Lem. 5.16].

Denote $S^{\circ }{:}{=}S \cap f^{-1}(U)$. By the decomposition theorem [4].^8^${\mathcal {IC}}_{U}$ is a direct summand of $R(f|_{S^{\circ }})_* {\mathbb {Q}}_{S^{\circ }}$. Taking stalks at *t*, we have

$$\begin{aligned} {\mathcal {H}}^0({\mathcal {IC}}_B)_t \simeq {\mathbb {Q}}_{B, t} \quad {\mathcal {H}}^i({\mathcal {IC}}_{U})_t \subseteq {\mathcal {H}}^i(R(f|_{S^{\circ }})_* {\mathbb {Q}}_{S^{\circ }})_t=0, \end{aligned}$$

because of the finiteness of $f|_{S^{\circ }}$. Thus, the natural map  is a quasi-isomorphism in the constructible derived category $D^b_c(B)$ with rational coefficients.

By the canonical bundle formula, there exists a ${\mathbb {Q}}$-divisor $\Delta \subset B$ such that the pair $(B, \Delta )$ is log terminal; see [43, Thm. 8.3.7.(4)] and [56, Thm. 2]. By the ${\mathbb {Q}}$-factoriality, *B* has log terminal singularities too. $\square$

#### Remark 1.11

(Quotient singularities) The finiteness of the restriction  over *b* suggests that *B* should have at worst quotient singularities. This would follow from the following conjecture.

#### Conjecture 1.12

[44, §2.24] Let  be a finite and dominant morphism from a smooth variety *X* onto a normal variety *Y*. Then *Y* has quotient singularities.

This is known for $n=2$ by [10, Lem. 2.6], but it is open in higher dimension. One of the main issue is that *f* itself need not be a quotient map, not even locally.

#### Corollary 1.13

The pullback  is injective.

#### Proof

By Proposition 1.10 this follows from the inclusion  coming from the decomposition theorem. $\square$

#### Remark 1.14

Let  be a surjective holomorphic map between compact complex manifolds, with *M* Kähler. By [70, Lem. 7.28], the pullback  is injective. However, this may fail if *N* is singular, e.g. if *f* is a normalization of a nodal cubic, even if *N* has ${\mathbb {Q}}$-factorial log terminal singularities, see for instance [52, Thm. 5.11].

#### Remark 1.15

Assume that *B* is projective. By Corollary 1.13, the smoothness of *B* can be dropped from the assumptions of Proposition 1.6 and Lemma 1.8, see also [50].

### The Fibers of a Fibration

Next we present Matsushita’s result that any fibration of a compact hyperkähler manifold is a Lagrangian fibration.

#### Lemma 1.16

(Matsushita) Assume  is a fibration. Then every smooth fiber $T{:}{=}X_t\subset X$ is a Lagrangian torus and in fact an abelian variety.

#### Proof

Comparing the coefficients of $x^{n-2}y^n$ in the polynomial (in *x* and *y*) the equation

$$\begin{aligned} q(\sigma +\bar{\sigma }+x\cdot \omega +y\cdot f^*\alpha )^n=c_X\cdot \int _X(\sigma +\bar{\sigma }+x\cdot \omega +y\cdot f^*\alpha )^{2n} \end{aligned}$$

shows $\int _X(\sigma \wedge \bar{\sigma })\wedge \omega ^{n-2}\wedge f^*(\alpha ^n)=0$ for all $\omega \in H^2(X,{\mathbb {R}})$ and all $\alpha \in H^2(B,{\mathbb {R}})$. Since $[T]=f^*(\alpha ^n)$ for some class $\alpha$, this implies $\int _F(\sigma \wedge \bar{\sigma })|_T\wedge \omega ^{n-2}|_T=0$, which for a Kähler class $\omega$ and using that $\sigma \wedge \bar{\sigma }$ is semi-positive implies $\sigma |_T=0$. Then conclude by Lemma 1.5. $\square$

#### Lemma 1.17

(Matsushita) The symplectic form $\sigma \in H^{2,0}(X)$ is trivial when restricted to any subvariety $T\subset X$ contracted to a point *t* under *f*. In particular,  all fibers of *f* are of dimension *n*,  i.e. *f* is equidimensional,  and if *B* is smooth,  *f* is flat.

#### Proof

A theorem due to Kollár [40, Thm. 2.1] and Saito [63, Thm. 2.3, Rem. 2.9.] says that $R^2f_*\omega _X$ is torsion free. Since in our case $\omega _X \simeq {\mathcal {O}}_X$, this shows that $R^2f_*{\mathcal {O}}_X$ is torsion free. Let $\bar{\sigma } \in H^2(X, {\mathcal {O}}_X)$ be the conjugate of the symplectic form, and $\rho$ be its image in $H^0(B, R^2f_*{\mathcal {O}}_X)$. Since the general fiber is Lagrangian, $\rho$ must be torsion and hence zero. If  is a resolution of *T*, then the image of $\bar{\sigma }$ in $H^2(\widetilde{T}, {\mathcal {O}}_{\widetilde{T}})$ is contained in the image ofand hence trivial. This implies that the image of $\sigma$ in $H^0(\widetilde{T}, \Omega ^2_{\widetilde{T}})$ is trivial, i.e. $\sigma |_T=0$. By semi-continuity of the dimension of the fibers, $\dim T \ge n$, and so *T* is Lagrangian.

The flatness follows from the smoothness of *X* and *B*, see [25, Exer. III.10.9].

$$\square $$

#### Remark 1.18

Note that the conclusion that *f* is flat really needs the base to be smooth. In fact, by miracle flatness, *f* is flat if and only if *B* is smooth.

### Further Results

We summarize a few further results without proof.

#### Higher Direct Images

The first one is the main result of [51].

##### Theorem 1.19

(Matsushita) Assume  is a fibration of a projective^9^ hyperkähler manifold over a smooth base. Then

$$\begin{aligned} R^if_*{{\mathcal {O}}}_X\simeq \Omega _B^i. \end{aligned}$$

On the open subset $B^{\circ } \subset B$ over which  is smooth, the result can be obtained by dualising the isomorphism

$$\begin{aligned} f^{\circ }_*\Omega ^1_{X^{\circ }/B^{\circ }} \simeq T_{B^{\circ }}, \end{aligned}$$

which holds because the smooth fibers of *f* are Lagrangian. A relative polarization is used to show that $R^1f^{\circ }_* {\mathcal {O}}_{X^{\circ }}$ and $f^{\circ }_*\Omega ^1_{X^{\circ }/B^{\circ }}$ are dual to each other. To extend the result from $B^{\circ }$ to the whole *B*, Theorem 1.19 uses a result of Kollár [40, Thm. 2.1] saying that $R^if_*\omega _X$ are torsion free, which for *X* hyperkähler translates into $R^if_*{{\mathcal {O}}}_X$ being torsion free.

As mentioned in Remark 1.7, the theorem implies $H^*(B,{\mathbb {Q}})\simeq H^*({\mathbb {P}}^n,{\mathbb {Q}})$.

#### Lagrangian Tori are Lagrangian Fibers

In [6] Beauville asked whether every Lagrangian torus $T\subset X$ is the fiber of a Lagrangian fibration . The question has been answered affirmatively:

(i) Greb–Lehn–Rollenske in [20] first dealt with the case of non-projective *X* and later showed in [21] the existence of an almost^10^ holomorphic Lagrangian fibration in dimension four.
(ii) A different approach to the existence of an almost holomorphic Lagrangian fibration with *T* as a fiber was provided by Amerik–Campana [1]. The four-dimensional case had been discussed before by Amerik [2].
(iii) Hwang–Weiss [33] deal with the projective case and proved the existence of an almost Lagrangian fibration with fiber *T*. Combined with techniques of [20] this resulted in a complete answer.

## Cohomology of the Base and Cohomology of the Fiber

The aim of this section is to prove the following result.

### Theorem 2.1

Assume  is a fibration and let $X_t$ be a smooth fiber. Then

The first isomorphism for *X* projective and *B* smooth is originally due to Matsushita [51], see Remark 1.7. The proof we give here is a version of the one by Shen and Yin [66] that works without assuming *X* projective. Note also that we do not assume that the base *B* is smooth.

The second isomorphism in degree two is essentially due to Oguiso [60], relying on results of Voisin [69]. The paper by Shen and Yin [66] contains two proofs of the general result, one using the ${{\mathfrak {s}}}{{\mathfrak {l}}}_2$-representation theory of the perverse filtration and another one, due to Voisin, relying on classical Hodge theory.

The proof we shall give avoids the perverse filtration as well as the various ${{\mathfrak {s}}}{{\mathfrak {l}}}_2\times {{\mathfrak {s}}}{{\mathfrak {l}}}_2$-actions central for the arguments in [66]. The discussion below also proves the second result in [66, Thm. 0.2], namely the equality

$$\begin{aligned} {^{\mathfrak {p}}}h^{i,j}(X)=h^{i,j}(X) \end{aligned}$$

between the classical and perverse Hodge numbers, see Sect. 2.3. How it fits into the setting of P $=$ W is explained in Sect. 3.

### Algebraic Preparations

To stress the purely algebraic nature of what follows we shall use the shorthand $H^*{:}{=}H^*(X,{\mathbb {C}})$ and consider it as a graded ${\mathbb {C}}$-algebra.

Consider a non-trivial, isotropic element $\beta$ of degree two, i.e. $0\ne \beta \in H^2$ with $q(\beta )=0$. Then, according to Verbitsky and Bogomolov [9, 68], one has

$$\begin{aligned} \beta ^n\ne 0\quad \text {and}\quad \beta ^{n+1}=0. \end{aligned}$$

In particular, multiplication by $\beta$ defines on $H^*$ the structure of a graded ${\mathbb {C}}[x]/(x^{n+1})$-algebra with *x* of degree two.

All that is needed in the geometric applications is then put into the following statement.

#### Proposition 2.2

For every two non-zero,  isotropic elements $\beta ,\beta '\in H^2,$ the induced graded ${\mathbb {C}}[x]/(x^{n+1})$-algebra structures on $H^*$ are isomorphic.

#### Proof

Consider the complex algebraic group of automorphisms $\mathrm{Aut}(H^*)$ of the graded ${\mathbb {C}}$-algebra $H^*$ and its image *G* under . Clearly, the assertion holds if $\beta ,\beta '\in H^2$ are contained in the same *G*-orbit. As any two non-zero isotropic classes $\beta ,\beta '$ are contained in the same orbit of the complex special orthogonal group $\mathrm{SO}(H^2,q)$, it suffices to show that $\mathrm{SO}(H^2,q)\subset G$. This follows from [65, Prop. 3.4], up to taking complex coefficients in loc. cit. $\square$

#### Remark 2.3

The arguments can be adapted to prove the following statement: Assume $\beta ,\beta '\in H^2$ satisfy $q(\beta )=q(\beta ')\ne 0$. Then the induced graded ${\mathbb {C}}[x]/(x^{2n+1})$-algebra structures on $H^*$, given by letting *x* act by multiplication with $\beta$ resp. $\beta '$, are isomorphic.

For $0\ne \beta \in H^2$ with $q(\beta )=0$ and $d\le n$ we letwhich is called the space of $\beta$-primitive forms. Note, however, that $\beta$ does not satisfy the Hard Lefschetz theorem; otherwise we would have defined primitive classes in $H^d$ as elements in the kernel of $\beta ^{2n-d+1}$.

We will also need the two spaces2.1It turns out that the map in the definition of $P_0$ is injective, but this is not needed for the argument. Note that $P_0H^d\subset \mathrm{Ker}(\beta ^n)\subset H^d$ for all $d>0$.

#### Corollary 2.4

The dimensions of the spaces $P_0H^d$ and ${\bar{P}}_0H^d$ are independent of the choice of the non-trivial,  isotropic class $\beta \in H^2$. $\square$

### Geometric Realizations

Let us begin by looking at the obvious choice for $\beta$ provided by the symplectic form $\sigma \in H^0(X,\Omega _X^2)\subset H^2(X,{\mathbb {C}})$.

#### Lemma 2.5

For $\beta =\sigma$ one has

$$\begin{aligned} P_0H^d=H^0(X,\Omega _X^d)\subset H^d(X,{\mathbb {C}})\quad \text {and}\quad P_0H^*\simeq H^*({\mathbb {P}}^n,{\mathbb {C}}) \end{aligned}$$

and

$$\begin{aligned} {\bar{P}}_0 H^d\simeq H^d(X,{{\mathcal {O}}}_X)\quad \text {and}\quad {\bar{P}}_{0} H^*\simeq H^*({\mathbb {P}}^n,{\mathbb {C}}). \end{aligned}$$

#### Proof

Concerning the first equality, one inclusion is obvious: Since $H^0(X,{{\mathcal {O}}}_X)=H^0(X,{\mathbb {C}})=H^0_{\sigma \text {-pr}}$, we have $H^0(X,\Omega _X^{d})={\mathbb {C}}\cdot \sigma ^{d/2}\subset P_0H^{d}$ for *d* even and $H^0(X,\Omega _X^d)=0$ for *d* odd. For the other direction, use that , for $p\le n$, is an isomorphism and that, therefore, for $q>0$ the composition2.2is injective. Hence, for $d\le n$, we have $\sigma ^{n-d+1}$ is injective, i.e. $H^{p,q}(X)\cap H^d_{\sigma \text {-pr}}=0$ for $q>0$, which is enough to conclude.

For the second part observe that $\mathrm{Ker}(\sigma ^n)\cap \bigoplus H^{p,q}(X)= \bigoplus _{p>0} H^{p,q}(X)$. $\square$

As an immediate consequence of Corollary 2.4 one then finds.

#### Corollary 2.6

For any non-trivial,  isotropic class $\beta \in H^2$ there exist isomorphisms

$$\begin{aligned} P_0H^*\simeq H^*({\mathbb {P}}^n,{\mathbb {C}})\quad \text {and}\quad {\bar{P}}_0H^*\simeq H^*({\mathbb {P}}^n,{\mathbb {C}}) \end{aligned}$$

of graded vector spaces. $\square$

Next let us consider a Lagrangian fibration . We consider the class $\beta {:}{=}f^*\alpha$, which is isotropic since $\alpha ^{n+1}=0$ for dimension reasons.

#### Lemma 2.7

For $\beta =f^*\alpha$ there exists an inclusion

$$\begin{aligned} f^*H^*(B,{\mathbb {C}})\subset P_0H^*(X,{\mathbb {C}}). \end{aligned}$$

#### Proof

The assertion follows from the Lefschetz decomposition

$$\begin{aligned} H^d(B, {\mathbb {C}})=I\!H^d(B,{\mathbb {C}})=\bigoplus _i\alpha ^i\cdot I\!H^{d-2i}(B,{\mathbb {C}})_\mathrm{pr} \end{aligned}$$

on *B*, with respect to the unique ample class $\alpha \in H^2(B,{\mathbb {Q}})$, see [13, Thm. 2.2.3.(c)], and the observation that pull-back via *f* maps $I\!H^{d-2i}(B,{\mathbb {C}})_\mathrm{pr}$ into $H^{d-2i}_{\beta \text {-pr}}$. $\square$

Corollary 2.4 then immediately implies

$$\begin{aligned} H^*(B,{\mathbb {C}})\simeq P_0H^*\simeq H^*({\mathbb {P}}^n,{\mathbb {C}}), \end{aligned}$$

see Remark 1.7, which proves the first part of Theorem 2.1.

We keep the isotropic class $\beta =f^*\alpha$ and observe that the natural inclusion2.3is actually an isomorphism.

#### Lemma 2.8

(Voisin) Let $\beta =f^*\alpha$ be as before and let $X_t\subset X$ be a smooth fiber of *f*. Then

#### Proof

The result is proved in [66, App. B]. The assertion is shown to be equivalent to the statement that the intersection pairing on the fiber is non-degenerate on the image of the restriction map, which in turn is deduced from Deligne’s global invariant cycle theorem. $\square$

From the result one obtains a surjectionSince ${\bar{P}}_{0}H^*\simeq H^*({\mathbb {P}}^n,{\mathbb {C}})$ by Corollary 2.6, its image in $H^*(X_t,{\mathbb {C}})$ is the subring generated by the restriction of a Kähler class. Hence, $\pi$ is an isomorphism, which proves the second isomorphism in Theorem 2.1. However, it is easier to argue directly, as the equality holds in Lemma 2.8 by (2.3).

### Perverse = Hodge

As in Sect. 2.1, we consider the abstract algebraic situation provided by $H^*{:}{=}H^{*}(X,{\mathbb {C}})$ and the additional structure induced by the choice of a non-zero isotropic class $\beta \in H^2$. The two spaces $P_0H^d$ and ${\bar{P}}_0H^d$ defined there, both depending on $\beta$, are part of a filtration

$$\begin{aligned} P_0H^*\subset P_1H^*\subset \cdots \subset P_{2n-1}H^*\subset P_{2n}H^*=H^*, \end{aligned}$$

where $P_0H^d$ is as defined before and ${\bar{P}}_0H^d=H^d/P_{d-1}H^d$.

In general, one defines2.4If we want to stress the dependence of $\beta$, we write $P_k^\beta H^d$. The graded objects of this filtration

$$\begin{aligned} \mathrm {Gr}_i^PH^*{:}{=}P_iH^*/P_{i-1}H^*, \end{aligned}$$

in particular $\mathrm {Gr}_dH^d={\bar{P}}_0H^d$, are used to define the *Hodge numbers* of the filtration as

$$\begin{aligned} {^{P}\!}h^{i,j}{:}{=}\dim \mathrm {Gr}_i^PH^{i+j}. \end{aligned}$$

As a further consequence of Proposition 2.2, one has

#### Corollary 2.9

The Hodge numbers ${^{P}\!}h^{i,j}$ of the filtration $P_iH^*$ are independent of the choice of the isotropic class $\beta \in H^2$. $\square$

Let us quickly apply this to two geometric examples.

(i) First, consider $\beta =\bar{\sigma }\in H^2(X,{{\mathcal {O}}}_X)\simeq H^{0,2}(X)\subset H^2(X,{\mathbb {C}})$, the anti-holomorphic symplectic form. Then the filtration gives back the Hodge filtration, i.e.
  $$\begin{aligned} P_k^{\bar{\sigma }}H^d=\bigoplus _{p\le k}H^{p,d-p}(X). \end{aligned}$$
  To see this, one needs to use the Lefschetz decomposition with respect to $\bar{\sigma }$:
  $$\begin{aligned} H^q(X,\Omega _X^p)=\bigoplus _{q-\ell \ge (q-n)^+}{\bar{\sigma }}^{q-\ell }\cdot H^{2\ell -q}(X,\Omega _X^p)_{\bar{\sigma }\text {-pr}}. \end{aligned}$$
  Note that from this example one can deduce that indeed for any choice of $\beta$ one has $P_k^\beta H^d=0$ for $k<0$ and $P_k^\beta H^d=H^d$ for $k\ge d$.
(ii) For the second example consider a Lagrangian fibration  and let $\beta$ be the pull–back of an ample class $\alpha \in H^2(B,{\mathbb {Q}})$. The induced filtration is called the *perverse filtration*^11^ and the Hodge numbers are denoted by ${^{\mathfrak {p}}}h^{i,j}(X)$.

Then [66, Thm. 0.2] becomes the following immediate consequence of Proposition 2.2 or Corollary 2.9.

#### Corollary 2.10

(Shen–Yin) For any Lagrangian fibration  the Hodge numbers of the perverse filtration equal the classical Hodge numbers : 

$$\begin{aligned} {^{\mathfrak {p}}}h^{i,j}(X)=h^{i,j}(X). \end{aligned}$$

## P $=$ W

P $=$ W for compact hyperkähler manifolds asserts that the perverse filtration associated with a Lagrangian fibration can be realised as the weight filtration of a limit mixed Hodge structure of a degeneration of compact hyperkäher manifolds. It boils down to the observation that the cup product by a semiample not big class and a logarithmic monodromy operator define nilpotent endomorphisms in cohomology which are not equal, but up to renumbering induce the same filtration.

Inspired by P $=$ W, we provide some geometric explanation or conjecture concerning the appearance of the cohomology of ${\mathbb {P}}^n$ in the introduction and in Theorem 2.1.

### The Weight Filtration of a Nilpotent Operator

#### Definition 3.1

Given a nilpotent endomorphism *N* of a finite dimensional vector space $H^*$ of index *l*, i.e. $N^{l}\ne 0$ and $N^{l+1}= 0$, the *weight filtration of* *N* *centered at* *l* is the unique increasing filtration

$$\begin{aligned} W_0H^*\subset W_1H^*\subset \cdots \subset W_{2l-1}H^*\subset W_{2l}H^*=H^*, \end{aligned}$$

with the property that (1) $N W_k \subseteq W_{k-2}$, and denoting again by *N* the induced endomorphism on graded pieces, (2) $N^k :\mathrm {Gr}^W_{l+k} H^* \simeq \mathrm {Gr}^W_{l-k}H^*$ for every $k\ge 0$, see [16, §1.6].

The weight filtration of *N* on $H^*$ can be constructed inductively as follows: first let $W_0{:}{=}{\mathrm {Im}} N^{l}$, and $W_{2l-1}{:}{=}\ker N^{l}$. We can replace $H^*$ with $W_{2l-1}/W_0$, on which *N* is still well-defined and $N^l=0$. Then define

$$\begin{aligned} W_1&{:}{=}\text {inverse image in }W_{2l-1}\text { of }{{\,\mathrm{Im}\,}}N^{l-1}\text { in }W_{2l-1}/W_0,\\ W_{2l-2}&{:}{=}\text {inverse image in }W_{2l-1}\text { of }{{\,\mathrm{Ker}\,}}N^{l-1}\text { in }W_{2l-1}/W_0. \end{aligned}$$

Continuing inductively, we obtain the unique (!) filtration on $H^*$ satisfying (1) and (2).

By the Jacobson–Morozov theorem, the nilpotent operator *N* can be extended to an $\mathfrak {sl}_2$-triple with Cartan subalgebra generated by an element $H^{N}$ which is unique up to scaling. By the representation theory of $\mathfrak {sl}_2$-triples, there exists a decomposition

$$\begin{aligned}H^*=\bigoplus ^{l}_{\lambda =-l} H^*_{\lambda },\end{aligned}$$

called the *weight decomposition*, with the property that $H^N(v)=\lambda v$ for all $v \in H^*_{\lambda }$. In particular, the decomposition splits the weight filtration of *N*

$$\begin{aligned}W_kH^*=\bigoplus ^{l}_{\lambda =l-k} H^*_{\lambda }.\end{aligned}$$

let us apply this to some geometric examples.

(i) Any cohomology class $\omega \in H^2(X, {\mathbb {C}})$ defines a nilpotent operator $L_{\omega }$ on $H^*{:}{=}H^*(X, {\mathbb {C}})$ by cup product. If $\omega$ is Kähler, then the Hard Lefschetz theorem implies that the weight filtration of $L_{\omega }$ on $H^*$ centered at 2*n* is^12^
  $$\begin{aligned} W^{\omega }_k H^*= \bigoplus _{i\ge 4n-k} H^{i}(X, {\mathbb {C}}). \end{aligned}$$
(ii) Consider a Lagrangian fibration  and let $\beta$ be the pull–back of an ample class $\alpha \in H^2(B,{\mathbb {Q}})$. Up to renumbering, the weight filtration associated with the class $\beta$ on $H^*$ centered at *n* coincides with the perverse filtration, see Sect. 2.3
  $$\begin{aligned}W^{\beta }_k H^d(X, {\mathbb {Q}})=P_{d+k-2n}H^{d}(X, {\mathbb {Q}}).\end{aligned}$$
  Indeed, the action of $\beta$ gives the morphisms  The isomorphism is called the *perverse Hard Lefschetz theorem* [13, Prop. 5.2.3]. By Proposition 2.2, this corresponds to the isomorphism $\bar{\sigma }^j:H^{n-j}(X, \Omega _X^i) \simeq H^{n+j}(X, \Omega _X^i)$.
(iii) Let  be a projective degeneration of hyperkähler manifolds over the unit disk which we assume to be semistable, i.e. the central fiber ${\mathcal {X}}_0$ is reduced with simple normal crossings. For $t \in \Delta ^*$, let *N* denote the logarithmic monodromy operator on $H^{*}({\mathcal {X}}_t, {\mathbb {Q}})$. The weight filtration of *N* centered at *d* on $H^d({\mathcal {X}}_t, {\mathbb {Q}})$, denoted by $W_{k}H^d({\mathcal {X}}_t, {\mathbb {Q}})$, is the weight filtration of the limit mixed Hodge structure associated to $\pi$, see [62, Thm. 11.40].

The degeneration  is called of type III if $N^{2} \ne 0$ and $N^{3}=0$ on $H^2({\mathcal {X}}_t, {\mathbb {Q}})$. In this case, the limit mixed Hodge structure is of Hodge–Tate type by [64, Thm. 3.8], and in particular $\mathrm {Gr}^W_{2i+1} H^{*}({\mathcal {X}}_t, {\mathbb {Q}})=0$. Then the even graded pieces of the weight filtration are used to define the *Hodge numbers*

$$\begin{aligned}{^{\mathfrak {w}}}h^{i,j}({\mathcal {X}}){:}{=}\dim \mathrm {Gr}^W_{2i} H^{i+j}({\mathcal {X}}_t, {\mathbb {Q}}).\end{aligned}$$

The Hodge numbers ${^{\mathfrak {w}}}h^{0,j}({\mathcal {X}})$ have a clear geometric description. The dual complex of ${\mathcal {X}}_0 = \sum \Delta _i$, denoted by $D({\mathcal {X}}_0)$, is the CW complex whose *k*-cells are in correspondence with the irreducible components of the intersection of $(k+1)$ divisors $\Delta _i$. The Clemens–Schmid exact sequence then gives

3.1 $$\begin{aligned} {^{\mathfrak {w}}}h^{0,j}({\mathcal {X}})=\dim H^{j}(D({\mathcal {X}}_0), {\mathbb {Q}}), \end{aligned}$$

see for instance [54, §3, Cor. 1 & 2].

In order to show P $=$ W, namely that the filtrations (ii) and (iii) can be identified, we need the notion of hyperkähler triples with their associated $\mathfrak {so}(5, {\mathbb {C}})$-action.

### Hyperkähler Triples

A hyperkähler manifold is a Riemannian manifold (*X*, *g*) which is Kähler with respect to three complex structures *I*, *J*, and *K*, satisfying the standard quaternion relations $I^2=J^2=K^2=IJK=-{\mathrm {Id}}$. The corresponding hyperkähler triple is the triple of Kähler classes in $H^2(X, {\mathbb {C}})\times H^2(X, {\mathbb {C}})\times H^2(X, {\mathbb {C}})$ given by

$$\begin{aligned}(\omega _I, \omega _J, \omega _K){:}{=}(g(I \cdot , \cdot ), g(J \cdot , \cdot ), g(K \cdot , \cdot )).\end{aligned}$$

The set of all hyperkähler triples on *X* forms a Zariski-dense subset in

$$\begin{aligned}D^{\circ }=\{(x,y,z)\mid \, q(x)=q(y)=q(z)\ne 0, q(x,y)=q(y,z)=q(z,x)=0\}.\end{aligned}$$

In particular, all algebraic relations that can be formulated for triples in $D^{\circ }$ and which hold for triples of the form $(\omega _I , \omega _J, \omega _K)$ hold in fact for all $(x, y, z) \in D^{\circ }$, see [66, Prop. 2.3].

### The ${\mathfrak {so}}(5, {\mathbb {C}})$-Action

Recall the scaling operatorBy the Jacobson–Morozov theorem, to any $\omega \in H^2(X, {\mathbb {C}})$ of Lefschetz type we can associate a $\mathfrak {sl}_2$-triple $(L_{\omega }, H, \Lambda _{\omega })$. Let $p=(x,y,z) \in D^{\circ }$. The $\mathfrak {sl}_2$-triples associated to *x*, *y* and *z* generate the Lie subalgebra $\mathfrak {g}_{p} \subset {\mathrm {End}}(H^*(X, {\mathbb {C}}))$, isomorphic to $\mathfrak {so}(5, {\mathbb {C}})$, with Cartan subalgebra

3.2 $$\begin{aligned} \mathfrak {h}=\langle H, H'_{p} {:}{=}\sqrt{-1}[L_y,\Lambda _z]\rangle . \end{aligned}$$

There is an associated weight decomposition

3.3 $$\begin{aligned} H^*(X, {\mathbb {C}})=\bigoplus _{i,j}H^{i,j}(p) \end{aligned}$$

such that for all $v\in H^{i,j}(p)$ we have

$$\begin{aligned} H(v)=(i+j-2n)v \quad H'_p(v)=(j-i)v. \end{aligned}$$

The following $\mathfrak {sl}_2$-triples in $\mathfrak {g}_p$

3.4 $$\begin{aligned}&E_p{:}{=}\frac{1}{2}(L_y-\sqrt{-1}L_z) \quad F_p{:}{=}\frac{1}{2}(\Lambda _y+\sqrt{-1}\Lambda _z) \quad H_{p}{:}{=}\frac{1}{2}(H+H'_p), \end{aligned}$$

3.5 $$\begin{aligned}&E'_p {:}{=}[E_p, \Lambda _x] \quad F'_p{:}{=}[L_x, F_p] \quad H'_p \end{aligned}$$

induce the same weight decomposition, since for any $v \in H^{i,j}(p)$ we have

$$\begin{aligned} H_p(v)=(j-n)v \quad H'_{p}(v)=(j-i)v. \end{aligned}$$

#### Remark 3.2

The previous identities for hyperkähler triples are due to Verbitsky. The result for a general triple $p=(x,y,z) \in D^{\circ }$ follows from the density of hyperkähler triples in $D^{\circ }$, and the fact that the $\mathfrak {sl}_2$-representation $H^*(X, {\mathbb {C}})$ associated to *x*, *y* and *z* have the same weights, since *x*, *y*, and *z* are all of Lefschetz type, see [66, §2.4].

### P $=$ W

The main result of [30] is the following.

#### Theorem 3.3

(P = W) For any Lagrangian fibration  there exists a type III projective degeneration of hyperkähler manifolds  with ${\mathcal {X}}_t$ deformation equivalent to *X* for all $t \in \Delta ^*,$ together with a multiplicative isomorphism $H^*(X, {\mathbb {Q}}) \simeq H^*({\mathcal {X}}_t, {\mathbb {Q}}),$ such that

$$\begin{aligned} P_kH^*(X, {\mathbb {Q}})=W_{2k}H^*({\mathcal {X}}_t, {\mathbb {Q}})=W_{2k+1}H^*({\mathcal {X}}_t, {\mathbb {Q}}). \end{aligned}$$

#### Proof

Let $\beta = f^*\alpha$ be the pullback of an ample class $\alpha \in H^2(B, {\mathbb {Q}})$, and $\eta \in H^2(X, {\mathbb {Q}})$ with $q(\eta )>0$. Since $\beta ^{n+1}=0$, we have $q(\beta )=0$. Up to replacing $\eta$ with $\eta + \lambda \beta$ for some $\lambda \in {\mathbb {Q}}$, we can suppose that $q(\eta )=0$. Set

$$\begin{aligned}y=\beta + \eta \quad z=-\sqrt{-1}(\eta -\beta ). \end{aligned}$$

By scaling a nonzero vector $x \in H^2(X,{\mathbb {C}})$ perpendicular to *y* and *z* with respect to *q*, we obtain $p({f}) = (x,y,z)\in D^{\circ }$ with

$$\begin{aligned} \beta = \frac{1}{2}(y - \sqrt{-1}z).\end{aligned}$$

Soldatenkov showed that the nilpotent operator $E'_{p(f)}$ is the logarithmic monodromy *N* of a projective type III degeneration  of compact hyperkähler manifolds deformation equivalent to *X*, see [64, Lem. 4.1, Thm. 4.6].^13^

The weight decomposition for the $\mathfrak {sl}_2$-triple (3.4) splits the perverse filtration associated to *f*, since $E_{p(f)}$ acts in cohomology via the cup product by $\beta$. The weight decomposition for the $\mathfrak {sl}_2$-triple (3.5) splits the weight filtration of the limit mixed Hodge structure associated to $\pi$, because $E'_{p(f)}=N$. Hence, by Sect. 3.3, this implies P $=$ W. $\square$

P $=$ W also provides alternative proofs of Corollary 2.10 and Theorem 2.1.

#### Corollary 3.4

(Numerical P = W) $${^\mathfrak {p}}h^{i,j}(X)={^\mathfrak {w}}h^{i,j}({\mathcal {X}}) =h^{i,j}(X)$$.

#### Proof

By Theorem 3.3 we obtain ${^{\mathfrak {p}}}h^{i,j}(X)={^{\mathfrak {w}}}h^{i,j}({\mathcal {X}})$. The equality ${^{\mathfrak {p}}}h^{i,j}(X)=h^{i,j}(X)$ is Corollary 2.10.

Alternatively, one can argue as follows. By [64, Thm. 3.8], the limit mixed Hodge structure $(H^{*}_{\mathrm {lim}}({\mathcal {X}}_t, {\mathbb {Q}})\simeq H^*({\mathcal {X}}_t, {\mathbb {C}}), W_*, F_*)$ associated to $\pi$ is of Hodge–Tate type, and so ${^{\mathfrak {w}}}h^{i,j}({\mathcal {X}})=\dim _{{\mathbb {C}}} \mathrm {Gr}^F_i H^{i+j}_{\mathrm {lim}}({\mathcal {X}}_t, {\mathbb {C}}).$ By the classical result [62, Cor. 11.25], we have $\dim _{{\mathbb {C}}} \mathrm {Gr}^F_i H^{i+j}_{\mathrm {lim}}({\mathcal {X}}_t, {\mathbb {C}})=h^{i,j}({\mathcal {X}}_t).$ We conclude that ${^{\mathfrak {p}}}h^{i,j}(X)=h^{i,j}({\mathcal {X}}_t)=h^{i,j}(X)$. $\square$

#### Corollary 3.5

At the boundary of the Hodge diamond of *X*,  P $=$ W gives^14^

In the following, we provide conjectural conceptual explanations for these identities.

### A Conjectural Explanation I

Assume that ${\mathcal {X}}$ is Calabi–Yau. This can be always achieved via a MMP, at the cost of making ${\mathcal {X}}_0$ mildly singular (precisely divisorial log terminal), see [18]. Under this assumption the homeomorphism class of $D({\mathcal {X}}_0)$ is well-defined.

Then the SYZ conjecture predicts that ${\mathcal {X}}_t$ carries a special Lagrangian fibration  with respect to a hyperkähler metric. By hyperkähler rotation [28, §3], *f* should become a holomorphic Lagrangian fibration  on a hyperkähler manifold *X* deformation equivalent to ${\mathcal {X}}_t$. It is conjectured that the base of a Lagrangian fibration on *X* is a projective space. So in brief, we should have the homeomorphisms

3.6 $$\begin{aligned} D({\mathcal {X}}_0) \simeq {\mathbb {P}}^{n} \simeq B. \end{aligned}$$

The latter equality is known to hold if $n\le 2$ , see Sect. 1.3, or conditional to the smoothness of the base [34]. The former equality is known for degenerations of Hilbert schemes or generalised Kummer varieties [8]. In both case, the most delicate problem is to assess the smoothness of $D({\mathcal {X}}_0)$ or *B*. From this viewpoint, the identity

$$\begin{aligned} \dim H^{j}(D({\mathcal {X}}_0), {\mathbb {Q}})=\dim H^j({\mathbb {P}}^n, {\mathbb {Q}})= \dim I\!H^j(B, {\mathbb {Q}})=\dim H^j(B, {\mathbb {Q}}). \end{aligned}$$

is a weak cohomological evidence for the conjecture (3.6).

### A Conjectural Explanation II

We conjecture that the equality ${^{\mathfrak {p}}}h^{i,0}({\mathcal {X}})={^{\mathfrak {w}}}h^{i,0}({\mathcal {X}})$ is the result of the identification of two Lagrangian tori up to isotopy.

#### Definition 3.6

Let *x* be a zero-dimensional stratum of ${\mathcal {X}}_0$. Choose local coordinates $z_0, \ldots , z_{2n}$ centered at *x* with $\pi (z)=z_0 \cdot \cdots \cdot z_{2n}$. For fixed radii $0 < r_i \ll 1$ and $t=\prod ^{2n}_{i=0}r_i$, a *profound torus* ${\mathbb {T}} \subset {\mathcal {X}}_t$ is

$$\begin{aligned} {\mathbb {T}} = \{ (r_0 e^{i \theta _0}, \ldots , r_{2n} e^{i \theta _{2n}})\mid \theta _0, \ldots , \theta _{2n} \in [0,2\pi ), \, \theta _0+ \cdots + \theta _{2n} - \arg (t)\in {\mathbb {Z}}\}. \end{aligned}$$

#### Remark 3.7

The ambient-isotopy type of ${\mathbb {T}} \subset {\mathcal {X}}_t$ does not depend on the choice of the coordinates: ${\mathbb {T}}$ is homotopic to $U_x \cap {\mathcal {X}}_t$, where $U_x$ is a neighbourhood of *x* in ${\mathcal {X}}$. More remarkably, if ${\mathcal {X}}$ is Calabi–Yau, then the isotopy class of ${\mathbb {T}}$ in ${\mathcal {X}}_t$ is independent of *x*. This follows at once from Kollár’s notion of ${\mathbb {P}}^1$-link (see [45, Prop. 4.37] or [26, Lem. 3.10]), or equivalently because profound tori are fibers of the same smooth fibration, by adapting [17, Prop. 6.12.]

#### Conjecture 3.8

(Geometric P = W) For any Lagrangian fibration  with general fiber *T*, there exists a projective minimal dlt type III degeneration of hyperkähler manifolds  with ${\mathcal {X}}_t$ deformation equivalent to *X* for all $t \in \Delta ^*,$ such that *T* is isotopic to a profound torus ${\mathbb {T}}$.

The conjecture is inspired by the geometric P $=$ W conjecture for character varieties, see the new version of [53] (to appear soon). Lemma 2.8 and (2.1) giveIf ${\mathcal {X}}_0$ has simple normal crossings (or dlt singularities modulo adapting [26, Thm. 3.12]), one obtains thatTherefore, Conjecture 3.8 would give a geometric explanation of P $=$ W at the highest weight

$$\begin{aligned}P_{d-1}H^d(X, {\mathbb {Q}}) = W_{2d-1}H^d({\mathcal {X}}_t, {\mathbb {Q}}).\end{aligned}$$

It is not clear what a geometric formulation of P $=$ W should be that could explain the cohomological statement in all weights.

Recent advance in the SYZ conjecture due to Li [48] suggests that profound tori can be made special Lagrangian, modulo a conjecture in non-archimedean geometry. A few months ago, the existence of a single special Lagrangian torus on ${\mathcal {X}}_t$ was a complete mystery, see [23, §5, p.152]. Note also that Li’s result is compatible with the expectation in symplectic geometry [3, Conj. 7.3]. Profound tori appear as general fibers of the SYZ fibration that Li constructed on an open set which contains an arbitrary large portion of the mass of ${\mathcal {X}}_t$ with respect to a Calabi–Yau metric, still modulo the non-archimedean conjecture. It is curious (but maybe not surprising) that also the previously quoted results [35] and [8] highly rely on non-archimedean techniques.

### Multiplicativity of the Perverse Filtration

P $=$ W implies that the perverse filtration on $H^*(X, {\mathbb {Q}})$ is compatible with cup product.

#### Corollary 3.9

(Multiplicativity of the perverse filtration) Assume $f:X \rightarrow B$ is a fibration. Then the perverse filtration on $H^*(X, {\mathbb {Q}})$ is multiplicative under cup product,  i.e.

#### Proof

By P=W, it is sufficient to show that the weight filtration is multiplicative. To this end, endow the tensor product $H^*({\mathcal {X}}_t, {\mathbb {Q}})\otimes H^*({\mathcal {X}}_t, {\mathbb {Q}})$ with the nilpotent endomorphism $N^{\otimes }{:}{=}N \otimes 1 + 1 \otimes N$, and call $W^{\otimes }$ the weight filtration of $N^{\otimes }$. Since the monodromy operator $e^{N}$ is an algebra homomorphism of $H^*({\mathcal {X}}_t, {\mathbb {Q}})$, *N* is a derivation, i.e.

$$\begin{aligned} N(x \cup y)= Nx\cup y + x \cup Ny= \cup (N^{\otimes }(x \otimes y)).\end{aligned}$$

As a consequence, the construction of the weight filtration (see Sect. 3.1) gives

$$\begin{aligned} \cup (W^{\otimes }_k (H^i({\mathcal {X}}_t, {\mathbb {Q}})\otimes H^j({\mathcal {X}}_t, {\mathbb {Q}})))\subseteq W_kH^{i+j}({\mathcal {X}}_t, {\mathbb {Q}}).\end{aligned}$$

Together with [16, 1.6.9.(i)] which says that

$$\begin{aligned}W^{\otimes }_k (H^i({\mathcal {X}}_t, {\mathbb {Q}})\otimes H^j({\mathcal {X}}_t, {\mathbb {Q}}))= \bigoplus _{a+b=k} W_a H^i({\mathcal {X}}_t, {\mathbb {Q}})\otimes W_b H^j({\mathcal {X}}_t, {\mathbb {Q}}), \end{aligned}$$

we conclude that the weight filtration is multiplicative. Alternatively see [30, §5].$\square$

#### Remark 3.10

For an arbitrary morphism of projective varieties or Kähler manifolds, the perverse filtration is not always multiplicative [71, Exa. 1.5], but it is so for instance if it coincides with the Leray filtration, or if P $=$ W holds. Indeed, the Leray filtration and the weight filtration of the limit mixed Hodge structure are multiplicative.

It is natural to ask whether the multiplicativity holds at a sheaf theoretic level, for $Rf_*{\mathbb {Q}}_X$, or over an affine base. The motivation for this comes from the celebrated P $=$ W conjecture for twisted character varieties [12], which has been proved to be equivalent to the conjectural multiplicativity of the perverse filtration of the Hitchin map that is a proper holomorphic Lagrangian fibration over an affine base, see [14, Thm. 0.6]. From this viewpoint, it is remarkable that Shen and Yin give a proof of the multiplicativity in the compact case [66, Thm. A.1] which uses only the representation theory of ${\mathfrak {sl}}(2)$-triples, with no reference to the weight filtration.

### Nagai’s Conjecture for Type III Degenerations

Let  be a projective degeneration of hyperkähler manifolds with unipotent monodromy $T_d$ on $H^d({\mathcal {X}}_t, {\mathbb {Q}})$. The *index of nilpotence* of $N_d{:}{=}\log T_d$ is

$$\begin{aligned} \mathrm {nilp}(N_d)=\max \{i \mid N^i_d\ne 0\}, \end{aligned}$$

and $\mathrm {nilp}(N_d) \le d$ by [22, Ch. IV]. It is known that $H^2({\mathcal {X}}_t, {\mathbb {Q}})$ determines the Hodge structure of $H^d({\mathcal {X}}_t, {\mathbb {Q}})$ by means of the LLV representation, see [65]. Nagai’s conjecture investigates to what extent $\mathrm {nilp}(N_2)$ determines $\mathrm {nilp}(N_d)$. The ring structure of the subalgebra generated by $H^2$ implies the inequality $\mathrm {nilp}(N_{2k})\ge k \cdot \mathrm {nilp}(N_{2})$, see [55, Lem. 2.4], but equality is expected.

#### Conjecture 3.11

(Nagai) $$\mathrm {nilp}(N_{2k})=k \cdot \mathrm {nilp}(N_{2})$$ for $$k \le 2n$$.

The previous inequalities imply Nagai’s conjecture for type III degenerations, i.e. $\mathrm {nilp}(N_{2})=2$. Remarkably, P $=$ W explains Nagai’s conjecture in terms of the level of the Hodge structure $H^d({\mathcal {X}}_t, {\mathbb {Q}})$, and determines $\mathrm {nilp}(N_d)$ even for *d* odd. Recall that the *level* of a Hodge structure $H = \oplus H^{p,q}$, denoted by $\mathrm {level}(H)$, is the largest difference $|p-q|$ for which $H^{p,q}\ne 0$, or equivalently the length of the Hodge filtration on *H*.

#### Proposition 3.12

Let  be a type III projective degeneration of hyperkähler manifolds with unipotent monodromy. Then

$$\begin{aligned} \mathrm {nilp}(N_d)=\mathrm {level}(H^d({\mathcal {X}}_t, {\mathbb {C}})). \end{aligned}$$

For $k\le 2n,$ the following identities hold : 

(i) $$\mathrm {nilp}(N_{2k}) =2k= k \cdot \mathrm {nilp}(N_{2}),$$
(ii) $$\mathrm {nilp}(N_{2k+1}) =2k-1,$$ if $$H^3({\mathcal {X}}_t, {\mathbb {C}})\ne 0$$.

#### Remark 3.13

The Statement (ii) is proved in [64, Prop. 3.15]. Here we present an alternative simple proof of (ii) which avoids the LLV representation.

Nagai’s conjecture is known to hold for degenerations of type I and III, i.e. for $\mathrm {nilp}(N_{2})=0$ and 2, see [37, Thm. 6.5]. In order to establish Nagai’s conjecture in full, only the case of type II degenerations remains open, i.e. when $\mathrm {nilp}(N_{2})=1$. For type II there are partial results: $k \le \mathrm {nilp}(N_{2k}) \le 2k-2$ for $2 \le k \le n-1$, see [37, Thm. 6.5], and $\mathrm {nilp}(N_{2n})=n$, see [31, Thm. 1.2]. The full conjecture holds for all the known deformation types of hyperkähler manifolds by [19, Thm. 1.13]. Further comments on Nagai’s conjecture for type II can be found in [19, 27, 31].

#### Proof

Let $l_d$ be half of the length of the weight filtration of $N_d$, i.e. $l_d{:}{=}\min \{i :W_{2i} H^d({\mathcal {X}}_t, {\mathbb {Q}}) = H^d({\mathcal {X}}_t, {\mathbb {Q}})\}.$ By Definition 3.1, we have $\mathrm {nilp}(N_d)=l_d$.

For any type III degeneration of Hodge structures of hyperkähler type with unipotent monodromy, we know by the proof of Theorem 3.3 that the logarithmic monodromy $N_*$ is of the form $E'_{p}=[\beta , \Lambda _x]$ for some $\beta$ and *x* in $H^2(X, {\mathbb {Q}})$ with $q(\beta )=0$. Here, we use the assumption $b_2({\mathcal {X}}_t)\ge 5$, see [64, §4.1]. Then, by Corollaries 2.9 and 3.4, we have $l_d=\mathrm {level}(H^d({\mathcal {X}}_t, {\mathbb {C}}))$. Hence, $\mathrm {nilp}(N_d)=\mathrm {level}(H^d({\mathcal {X}}_t, {\mathbb {C}}))$.

Finally, statements (i) and (ii) are equivalent to (i) $H^{2k,0}({\mathcal {X}}_t)={\mathbb {C}}\sigma \ne 0$, and (ii) $H^{2k,1}({\mathcal {X}}_t) \ne 0$ if $H^{2,1}({\mathcal {X}}_t) \ne 0$, which follows from (2.2). $\square$

## Examples and Counterexamples

### Example 4.1

In [57, Ex. 1.7.(iv)] Namikawa exhibits an example of a submanifold *T* of a hyperkähler manifold *X* which is isomorphic to a complex torus, but is not Lagrangian (actually it is symplectic). Let *E*, *F* be elliptic curves defined by the cubic equations *f* and *g* respectively, and let $Y \subseteq {\mathbb {P}}^5$ be the cubic fourfold given by the equation $h{:}{=}f(x_0,x_1,x_2)+g(y_0,y_1,y_2)=0$. The cyclic group $G {:}{=}{{\mathbb {Z}}}/3{{\mathbb {Z}}}$ acts on *Y* bywhere $\zeta$ is a primitive third root of unity. The induced action on the Fano variety of lines *X* is symplectic, i.e. $\phi _{\zeta }^*\sigma = \sigma$ for $\sigma \in H^0(X, \Omega ^2_X)$. Indeed, by [5] there is a *G*-equivariant isomorphism $H^0(X, \Omega ^2_X)\simeq H^1(Y,\Omega ^3_Y)$. Denoting by $\Omega$ the canonical section of $H^0({\mathbb {P}}^5, K_{{\mathbb {P}}^5}(6))$, $H^1(Y,\Omega ^3_Y)$ is generated by the *G*-invariant residue ${\mathrm {Res}}_Y(\Omega /h^2)$, and so the action is symplectic. In particular, the fixed locus *T* of the *G*-action on *X* is a symplectic submanifold. One defines *T* as the set of lines which join two points on $Y \cap \{y_0=y_1=y_2=0\} \simeq E$ and $Y \cap \{x_0=x_1=x_2=0\} \simeq F$ respectively. Hence, $T \simeq E \times F$. We conclude that *T* is a symplectic torus embedded in the hyperkähler manifold *X*.

### Example 4.2

There exists a Lagrangian submanifold *L* of a hyperkähler manifold *X* with

### Proof

Let  be an elliptic K3 surface with smooth fiber *E*. Define $L \subseteq X {:}{=}S^{[2]}$ to be the locus of non-reduced length-two subschemes of *S* supported on *E*, which is isomorphic to the ${\mathbb {P}}^1$-bundle ${\mathbb {P}}(\Omega ^1_{S}|_E)$ over *E*. Then, *L* is an irreducible component of the fiber of the Lagrangian fibration , thus *L* is Lagrangian. The exceptional divisor $\mathrm Exc$ of the Hilbert–Chow morphism  restricts to a multiple of the tautological line bundle ${\mathcal {O}}_{{\mathbb {P}}(\Omega ^1_{S}|_E)}(-1)$ on *L*. Therefore, the second cohomology group $H^2(L)$ is generated by the restriction of $\mathrm Exc$ and the pullback of an ample line bundle of $S^{(2)}$. $\square$

### Example 4.3

There exists a Lagrangian submanifold *L* of a hyperkähler manifold *X* with

### Proof

Let *C* be a smooth curve of genus two in an abelian surface *A*. Consider the moduli space $M_{\text {odd}}(A)$ of stable 1-dimensional sheaves on *A* supported on the curve class

$$\begin{aligned}2[C] \in H_2(A, {{\mathbb {Z}}})\end{aligned}$$

and Euler characteristic $-1$. The fiber of the Albanese morphism  is a compact hyperkähler manifold *X* deformation equivalent to a generalised Kummer variety of dimension six. Taking Fitting supports defines a Lagrangian fibrationThe fiber over the curve 2*C* contains the locus *L* of stable sheaves ${\mathcal {F}}$ on *A* such that the composition  factors via the natural map . As ${\mathcal {O}}_{C}$-module, ${\mathcal {F}}$ is a rank-two vector bundle, and *L* can be identified with the moduli space of rank-two vector bundles on *C* of degree one, which is isomorphic to the intersection of two quadrics in ${\mathbb {P}}^5$, see [15, 59]. The cohomology $H^*(X)$ is generated by so-called tautological classes, and $H^*(L)$ is generated by their restrictions, see [49] and [58, Thm. 1]. Therefore, we have$\square$

## Footnotes

1 Here and in (iii) and (iv), one expects isomorphisms of ${\mathbb {Q}}$-algebras, but this seems not known.

2 Is there an a priori reason why this is the case for Lagrangian tori? It fails for general Lagrangian submanifolds; see Sect. 4.

3 The non-triviality of the scalar for $\mathrm{c}_2(X)$ follows from the fact that $\int _X\mathrm{c}_2(X)\cdot \omega ^{2n-2}\ne 0$ for any Kähler class $\omega$.

4 The traditional proof goes as follows: First one shows that for any non-trivial class $\alpha \in H^2(B,{\mathbb {R}})=H^{1,1}(B,{\mathbb {R}})$ and any Kähler class $\omega$ on *X* one has $\int _X(f^*\alpha )\wedge \omega ^{2n-1}\ne 0$. Indeed, otherwise the Hodge index theorem would imply $q(f^*\alpha )<0$ and, therefore, $(f^*\alpha )^{n+1}\ne 0$, which contradicts $\dim (B)=n$. As a consequence, observe that for any two non-trivial classes $\alpha _1,\alpha _2\in H^2(B,{\mathbb {R}})$ there exists a linear combination $\alpha {:}{=}\lambda _1\alpha _1+\lambda _2\alpha _2$ with $\int _X(f^*\alpha )\wedge \omega ^{2n-1}=0$, which then implies $\alpha =0$, i.e. any two classes $\alpha _1,\alpha _2\in H^2(B,{\mathbb {R}})$ are linearly dependent.

5 By Lemma 1.8 below, *B* is a Fano manifold, which provides an alternative argument of the simply connectedness of *B*.

6 By evoking results due to Saito [63], it should be possible to avoid the projectivity assumption in [41].

7 Are the singularities of *B* actually factorial?

8 Alternatively, note that the trace map  splits the natural morphism .

9 Again, the projectivity assumption can presumably be dropped by applying results of Saito.

10 A meromorphic map $f :X \dashrightarrow B$ is almost holomorphic if there exists a Zariski-open subset $U \subset B$ such that  is holomorphic and proper.

11 The classical definition of the perverse filtration for the constructible complex $Rf_*{\mathbb {Q}}_X$ due to [4] or [13, Def. 4.2.1] coincides with the present one; see [13, Prop. 5.2.4.(39)].

12 The equality actually holds for any Kähler manifold, not necessary hyperkähler.

13 One can use the Lie algebra structure of the LLV algebra to compare the present description of $E'_{p(f)}$ with that of [64, Lem. 4.1], see [46, Lem. 3.9]. Mind that Soldatenkov’s existence result is not constructive: it relies on lattice theory and the geometry of the period domain, and does not produce an explicit type III degeneration.

14 The identity $\dim H^{j}(D({\mathcal {X}}_0), {\mathbb {Q}})=\dim H^j({\mathbb {P}}^n)$ was first proved in [37, Thm. 7.13].
